# Importance of Considering Fed-State Gastrointestinal Physiology in Predicting the Reabsorption of Enterohepatic Circulation of Drugs

**DOI:** 10.1007/s11095-024-03669-3

**Published:** 2024-03-12

**Authors:** Kohei Nakamura, Atsushi Kambayashi, Satomi Onoue

**Affiliations:** 1 Pharmaceutical Research and Technology Labs, Astellas Pharma Inc., 21 Miyukigaoka, Tsukuba, Ibaraki 305-0841 Japan; 2 Faculty of Pharmaceutical Sciences, Tokyo University of Science, 2641 Yamazaki, Noda, Chiba 278-8510 Japan; 3 School of Pharmaceutical Sciences, University of Shizuoka, 52-1 Yada, Suruga-Ku, Shizuoka, 422-8526 Japan

**Keywords:** bile micelles, enterohepatic circulation, fed, *in silico* modeling and simulation, oral dosage forms

## Abstract

**Purpose:**

The purpose of this study was to develop a simulation model for the pharmacokinetics (PK) of drugs undergoing enterohepatic circulation (EHC) with consideration to the environment in the gastrointestinal tract in the fed state in humans. The investigation particularly focused on the necessity of compensating for the permeability rate constant in the reabsorption process in consideration of drug entrapment in bile micelles.

**Methods:**

Meloxicam and ezetimibe were used as model drugs. The extent of the entrapment of drugs inside bile micelles was evaluated using the solubility ratio of Fed State Simulated Intestinal Fluid version 2 (FeSSIF-V2) to Fasted State Simulated Intestinal Fluid version 2 (FaSSIF-V2). Prediction accuracy was evaluated using the Mean Absolute Percentage Error (MAPE) value, calculated from the observed and predicted oral PK profiles.

**Results:**

The solubilization of ezetimibe by bile micelles was clearly observed while that of meloxicam was not. Assuming that only drugs in the free fraction of micelles permeate through the intestinal membrane, PK simulation for ezetimibe was performed in both scenarios with and without compensation by the permeation rate constant. The MAPE value of Zetia® tablet, containing ezetimibe, was lower with compensation than without compensation. By contrast, Mobic® tablet, containing meloxicam, showed a relatively low MAPE value even without compensation.

**Conclusion:**

For drugs which undergo EHC and can be solubilized by bile micelles, compensating for the permeation rate constant in the reabsorption process based on the free fraction ratio appears an important factor in increasing the accuracy of PK profile prediction.

## Introduction

Enterohepatic circulation (EHC) is a circulatory process in which drugs and biological components absorbed into the body accumulate in the gallbladder and are excreted into the duodenum with bile acids at a fixed timing, then reabsorbed in the small intestine. The common characteristics of drugs undergoing EHC is that they are relatively large molecules and tend to be distributed into bile acids (estimated threshold is around 500 – 600 Da; smaller compounds can be eliminated via urine) [1]. Nevertheless, EHC is not controlled by molecular size only, given that EHC has been reported for many compounds below this threshold of molecular weight [2]. More than 100 compounds, including morphine and warfarin, have been reported to undergo EHC [1–5]. While the importance of accurate simulation of elimination processes in planning of therapeutic drug monitoring and estimating trough concentration is well understood, the complexity of these processes is increased for drugs undergoing EHC.

Given their distinctive multimodal pharmacokinetics (PK) profile, PK of compounds which undergo EHC must be predicted using simulation models that can incorporate EHC phenomena. Most simulation models for EHC apply a gallbladder compartment to typical 1- or 2-compartment models, in which drugs distribute to the gallbladder in a first-order manner [6, 7]. Drugs in the gallbladder are often described as excreted into the gastrointestinal tract (GIT) at the time of meal ingestion, and are then reabsorbed to the central compartment in a first-order absorption rate-consistent manner [6, 7]. A trigonometric function has also been used to describe the timing of gallbladder excretion unrelated to meals [8]. Ibarra *et al*. emphasized that drugs excreted to the GIT are not necessarily completely reabsorbed, and that the degree of reabsorption may depend on the characteristic of the individual drug [9]. Wang *et al*. analyzed the PK variability of mycophenolate mofetil – the prodrug of mycophenolic acid – as it is subject to EHC. Their study focused on the absorption process, and mentioned the importance of a predictive *in vitro* dissolution test which takes account of dynamics in the GIT, such as gastric emptying [10]. However, most modeling and simulation (M&S) for EHC to date appears to have focused on processes relating to systemic distribution and excretion from the gallbladder, with less focus on behavior in the GIT, particularly the reabsorption process.

One of the most important determinants of the extent and rate of drug reabsorption after emptying to the GIT in EHC appears to be those bile acids emptied from the gallbladder together with the drug. The main trigger for the onset of EHC is considered to be meal ingestion, specifically the post-prandial presence of fat components from the meal in the duodenum [11]. Accordingly, the GIT environment at the timing of EHC is the fed state. This state markedly differs from the fasted state, and is characterized by an abundance of bile acids. Primary bile acids in humans are taurine or glycine-conjugated cholic acid and chenodeoxycholic acid [12]. In the fed state in the small intestine, bile acids occurring at concentrations above the critical micelle concentration (CMC) form micelles [13] which can incorporate drug compounds within them, albeit that the extent of incorporation is highly dependent on the characteristics of the individual drug. It is considered that drug compound entrapped in micelles must be released from the micelles before it can permeate the epithelial cell membrane in the small intestine [14, 15]. Given this phenomenon, we considered that membrane permeability in the reabsorption process of EHC may differ from that in the fasted state. To our knowledge, however, no study which has simulated the PK profile of drugs undergoing EHC has considered the impact of the fed state on reabsorption.

**Fig. 1:**
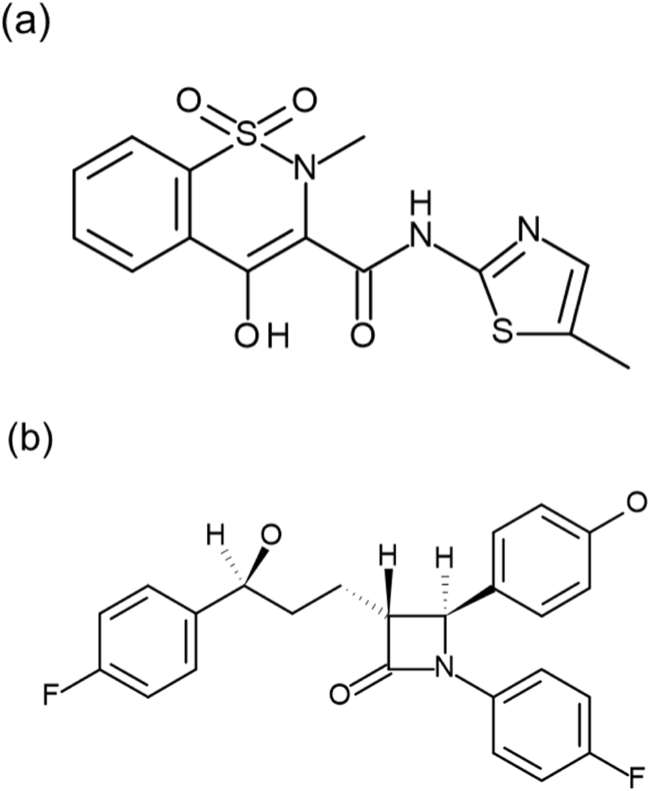
Chemical structure of (**a**) meloxicam and (**b**) ezetimibe.

The aim of this study was to evaluate the impact of the fed state on the PK prediction of EHC using M&S, with a particular focus on compensation of the permeability rate in consideration of drug entrapment in bile micelles. Kiyota *et al*. developed the M&S to predict the PK profile of oral dosage forms with integration of the transit and dissolution processes of the formulation in the GIT. They have used the M&S to successfully predict PK profiles in both prandial states [16, 17]. In the present study, we applied their model to PK prediction with consideration to the EHC reabsorption process. As model drugs we selected meloxicam and ezetimibe (Fig. 1), which are known to undergo EHC. Meloxicam is a non-steroidal anti-inflammatory drug (NSAID). An in silico EHC model of this drug after iv administration has been reported [18]. Ezetimibe is an inhibitor of Niemann-Pick C1-like 1 (NPC1L1) receptor [19]. It is mainly expressed on the brush border membrane in the small intestine and transports cholesterol to the blood circulation, and ezetimibe can reduce cholesterol concentration in the plasma by inhibiting absorption. Prediction models for ezetimibe have been reported [6, 20, 21]. Despite these characteristics, however, all the models for these two compounds in the literature have used the same absorption rate constant as that before the occurrence of EHC. The present study is the first to establish M&S for EHC considering not only the transit and dissolution of the formulation in the GIT but also the reabsorption process, with compensation for the permeability rate constant in the fed state.

## Materials and Methods

### Model Drugs

The physicochemical properties of meloxicam and ezetimibe are summarized in Table I. Both drugs are known to undergo EHC [6, 18, 20, 22]. To investigate the impact of the entrapment of drugs in bile micelles on reabsorption during EHC, we selected these drugs, with different physicochemical properties, as model drugs. Ezetimibe is a non-ionic form and likely has low solubility in the neutral pH of the intestine, and was selected based on the assumption that the entrapment of drugs in micelles happens due to the lipophilicity. In contrast, meloxicam is an ionic form and likely has high solubility at a neutral pH; for this drug, we had a lower expectation that the drug was entrapped in bile micelles.

**Table I:** Summary of physicochemical properties of meloxicam and ezetimibe

	Meloxicam	Refs	Ezetimibe	Refs
CAS number	71,125–38-7	-	163,222–33-1	-
Molecular formula	C14H13N3O4S2	-	C24H21F2NO3	-
Molecular weight	351.40	-	409.43	-
BCS	II	[23]	II	[24]
Log P	3.32	[25]	4.5	[26]
pKa	1.09, 4.18	[25]	9.7	[26]

Log P of meloxicam was a calculated value based on apparent partition coefficient Log P_app_ = 0.1 in n-octanol/buffer pH 7.4 [25]

### Materials

Mobic® tablet 10 mg (lot 289,004) was purchased from Boehringer Ingelheim GmbH (Ingelheim, Germany). Zetia® Tablet 10 mg (lot W025574) was purchased from Organon & Co. (Jersey City, NJ, USA). Drug powders of meloxicam (lot HSRPM-NQ) and ezetimibe (lot 3CDDL-BR) were purchased from Tokyo Chemical Industry Co., Ltd. (Tokyo, Japan). Acetonitrile, hydrochloric acid solution (1 mol/L), sodium perchlorate monohydrate, and perchloric acid were purchased from FUJIFILM Wako Pure Chemical (Osaka, Japan). Sodium chloride was purchased from Junsei Chemical Co., Ltd. (Tokyo, Japan). Maleic anhydride and sodium hydroxide solution (1 mol/L) were purchased from Kanto Chemical Co., Inc. (Tokyo, Japan). FaSSIF/FeSSIF/FaSSGF powder (lot FFF-0321-A), FaSSIF-V2 powder (lot V2FAS-0221-A), and FeSSIF-V2 powder (lot V2FES-0121-A) were purchased from Biorelevant.com Ltd. (London, United Kingdom).

### Methods

#### Preparation of Biorelevant Media

Fasted State Simulated Gastric Fluid (FaSSGF) and Fasted State Simulated Intestinal Fluid version 2 (FaSSIF-V2) were used for dissolution tests and solubility tests. Fed State Simulated Intestinal Fluid version 2 (FeSSIF-V2) was used for solubility tests. These media contain sodium taurocholate as the bile acid. Their composition and preparation method have been previously reported [13] and were prepared accordingly, except that FaSSGF was used without pepsin.

#### *In vitro* Dissolution Test and Solubility Measurement

For *in vitro* dissolution tests, a USP apparatus II paddle dissolution tester (NTR-6400AC, Toyama Sangyo Co., Ltd., Osaka, Japan) was used. The media volume of FaSSGF and FaSSIF-V2 was 300 mL and 500 mL, respectively, and temperature in the dissolution vessel was maintained at 37℃ ± 0.5℃. A Mobic® tablet or a Zetia® tablet was put in a vessel filled with the medium, and approximately 5 mL samples were withdrawn using a stainless-steel cannula and plastic syringe at 5, 10, 15, 30, 45, and 60 min. Paddle rotation speed during the dissolution test was maintained at 50 rpm for up to 60 min. The samples were filtered through PVDF 0.45 µm filters (Whatman, GD/X, 13 mm, Cytiva, Chicago, IL, USA) immediately after withdrawal, and filtrates were recovered after the first 2 mL was discarded. Filtrates of the Mobic® tablet in FaSSIF-V2 were diluted 10 times with acetonitrile while the other filtrates were mixed with the equal volume of acetonitrile. All dissolution tests were conducted in triplicate.

The solubility of meloxicam in Mobic® tablet and ezetimibe in Zetia® tablet in the biorelevant media for PK simulation were estimated from the infinity point of each dissolution test, in which paddle rotation speed was raised to 250 rpm for a further 60 min after the dissolution profile was obtained at 50 rpm for first 60 min. When the tablets dissolved completely during the infinity spin in the dissolution test, separate solubility measurements were performed, as described in the next paragraph.

In solubility studies using the drug substances, the excess amount of meloxicam powder was added to 10 mL blank FaSSIF-V2, FaSSIF-V2 and FeSSIF-V2. The excess amount of ezetimibe powder was added to 10 mL FaSSIF-V2 and FeSSIF-V2. Test tubes containing the drug substances in the biorelevant media were vigorously shaken at 37℃ ± 0.5℃ for 5 h. An approximately 5 mL sample was withdrawn using a plastic syringe, then filtered through a PVDF 0.45 µm filter. The filtrates (after discarding the first ca. 2 mL) were diluted with 50:50 v/v acetonitrile for analysis to prevent precipitation, except for that of ezetimibe tested in FeSSIF-V2. All solubility tests were conducted in triplicate.

The concentrations of meloxicam and ezetimibe in biorelevant media were quantified using an HPLC system (Alliance Separations Module 2695 with detector of type 2489, Waters Corporation, Milford, MA, USA). TSKgel ODS-100Z 5 µm (4.6 mm × 15 cm, Tosoh Corporation, Tokyo, Japan) was used as analytical column for both drugs. Column temperature during analysis was set to 40℃. A mixture of perchlorate (Na) buffer pH 2.5 and acetonitrile (50:50, v/v) was used as mobile phase. The wavelength of detection for meloxicam and ezetimibe was 354 nm and 232 nm, respectively. Flow rate and injection volume were 1.2 mL/min and 10 μL, respectively. The calibrated range of the calibration curve for meloxicam and ezetimibe was from 0.0001 mg/mL to 0.01 mg/mL under these analytical conditions.

#### *In silico* Modeling and Simulation

**Fig. 2:**
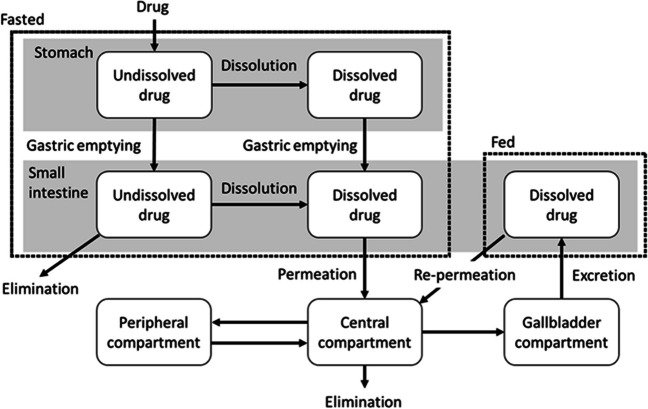
Outline of M&S considering EHC used in this study.

The outline of the model used in this study to predict the oral PK profile in human with consideration to EHC is shown in Fig. 2. We assumed that the drugs were orally administered to healthy adult subjects in the fasted state and that the fasting state continued for 4 h until a meal was served. The first absorption process of the administered formulation in the fasted state was described using a previously reported model (compartments and equations) [27]. To describe the EHC process, the drugs in the central compartment were assumed to not only be eliminated and distributed to the peripheral compartment but also to the gallbladder compartment with first-order kinetics. The drugs in the gallbladder compartment were then assumed to be excreted to the GIT (duodenum) at the timing of meal ingestion. The environment in the GIT when bile excretion occurred was assumed to be the fed state, in which the effective permeability (P_eff_) of drugs in the small intestine in humans was compensated for based on the extent of entrapment in the micelles estimated from the *in vitro* study. The gastrointestinal transit of fluid was separately described for the fasted and fed states.

The dissolution rate of drugs in the GIT (stomach and small intestine) and in the *in vitro* tests were assumed to follow the following equation [28]:

1 $$\frac{d{W}_{diss,t}}{dt}=z\cdot {{W}_{undiss,t}}^\frac{2}{3}\cdot \left({C}_{s}-\frac{{W}_{diss,t}}{{V}_{t}}\right)$$

where *W*_*diss,t*_ and *W*_*undiss,t*_ are the amount of dissolved and undissolved drugs, respectively, in the GIT or the dissolution vessel at time *t*; *z* is the dissolution rate constant; *V*_*t*_ is the fluid volume in the GIT (stomach or small intestine) or the dissolution vessel at time *t*; *C*_*s*_ is the saturated solubility of the drug in the stomach or the small intestine. The *z* value was estimated from the *in vitro* dissolution profile in the biorelevant media using Solver in Microsoft Excel (Microsoft, Redmond, WA, USA).

In the case that the dose used for the *in vitro* dissolution test differed from that administered to humans, the dissolution rate constant was compensated for using the following equation [29]:

2 $${z}_{vivo}={z}_{vitro}\cdot {\left(\frac{{D}_{vivo}}{{D}_{vitro}}\right)}^\frac{1}{3}$$

where *z*_*vitro*_ and *z*_*vivo*_ are the dissolution rate constants in the *in vitro* dissolution test and the *in vivo* GIT, respectively; and *D*_*vitro*_ and *D*_*vivo*_ are the dose used for the *in vitro* dissolution test and *in vivo* study, respectively.

Gastric emptying of the drug (dissolved and undissolved) and of gastric fluid in fasted humans was assumed to follow the first-order equation:

3 $$\frac{d{G}_{fasted,t}}{dt}=2.8\cdot {X}_{fasted,t}$$

where *G*_*fasted,t*_ is the amount of drug or fluid volume already emptied from the stomach at time *t*; and *X*_*fasted,t*_ is the amount of drug or fluid remaining in the stomach at time *t*. The gastric rate constant was set based on a report that the half gastric emptying time was 15 min [30]. The initial fluid volume in the stomach was set to 50 mL [31] and the ingested water volume was set to 150 mL, which was the volume administered in a human PK study of Zetia® tablet performed in Japan [32]; accordingly, the initial total volume in the stomach in the fasted state was set to 200 mL. Due to the absence of information on the volume of ingested water for the PK study of Mobic® tablet, the same initial volume as that of Zetia® tablet was assumed for Mobic® tablet.

In this study, the timing of the gallbladder release of drugs to the GIT was assumed to be that of meal ingestion. Meal timing was set to 4, 10, 24, 28, 34, 48, 52, 58 and 72 h after administration. The gastric fluid volume of 584 mL was added to the stomach fluid compartment at the timing of each meal ingestion in accordance with a previous report [33].

The emptying rate of gastric content in the fed state was assumed to follow these equations [16]:

4 $$\frac{d{G}_{fed,t}}{dt}=7.21\cdot ({X}_{fed,t}-500)$$

5 $$\frac{d{G}_{fed,t}}{dt}=0.452\cdot {X}_{fed,t}$$

Gastric emptying rate in the fed state was described as changing depending on the time elapsed after meal ingestion. This characteristic is the basis for the use of Eq. (4) (within 1.5 h after the last mealtime) and Eq. (5) (1.5 or more hours until the next mealtime). *G*_*fed,t*_ and *X*_*fed,t*_ in these equations are the amount of gastric content already emptied from the stomach and the amount of gastric content remaining in the stomach, respectively, at time *t*.

For the transit of gastric content in the fed state, it is necessary to consider not only the amount of water in the ingested food but also the secretion of gastric juice. The rate of gastric juice secretion was assumed to follow the following equation [16]:

6 $${Y}_{t}=540\cdot (1-exp\left(-{(t-{t}_{diet})}^{{~}^{-2.206}\!\left/ \!{~}_{2.553}\right.}\right))$$

where *Y*_*t*_ is the rate of gastric juice secretion at time *t*; and *t*_*diet*_ is time of ingestion of the preceding meal at time *t*.

The permeability rate of dissolved drugs through the small intestinal membrane in humans was assumed to follow Eq. (7):

7 $$\frac{d{A}_{t}}{dt}={P}_{eff}\cdot SA\cdot \frac{{W}_{t}}{{V}_{t}}\cdot f$$

where *A*_*t*_ is the amount of drugs already permeated through the small intestinal membrane at time *t*; P_eff_ is effective permeability in the intestine in humans; SA is the surface area of the small intestine in humans, which was set to 800 cm^2^ in this study [34]; *W*_*t*_ and *V*_*t*_ are the amount of drugs dissolved and fluid volume in the intestine, respectively, at time *t*; and *f* is the free fraction rate of drugs not entrapped in mixed micelles, which was calculated using the following equation [35]:

8 $$f=1-\left(\frac{{C}^{+}-{C}^{-}}{{C}^{+}}\right)$$

where C^+^ and C^−^ are the saturated solubility of drugs in FeSSIF-V2 and FaSSIF-V2, respectively.

Sodium taurocholate and lecithin concentration in FaSSIF-V2 are 3 mM and 0.2 mM, respectively, and those in FeSSIF-V2 are 10 mM and 2 mM, respectively [13]. Since micelles did not clearly form with a surfactant (taurocholate and lecithin) concentration of 2—3 mM in a previous study of CMC in FaSSIF-V2 [36], free fraction rate in this study was calculated from the solubility ratio of FeSSIF-V2 to FaSSIF-V2. The formulation was considered able to exist in the small intestine for 4 h after administration [37]. Initial intestinal fluid volume in both prandial states was set to 100 mL [31].

Since P_eff_ in the small intestine in humans for meloxicam and ezetimibe have not been reported, it was necessary to estimate them from the apparent permeability through Caco-2 cells (P_app_) [38]. The P_app_ for each compound was cited from previous reports [39, 40] or calculated using an equation [41] based on the reported partition coefficient [25, 42]. In addition, permeation of the unstirred water layer on the brush border of the intestinal epithelial cells appears to be an important step for the whole permeation process; the rate constant of this permeation (P_UWL_) can reportedly be estimated from the molecular weight using Eq. (9) [43]:

9 $${P}_{UWL}=10\cdot {10}^{-4}\cdot {\left(\frac{180}{MW}\right)}^\frac{1}{3}$$

where MW is molecular weight.

The estimated rate constants are summarized in Table II. Both compounds showed higher P_UWL_ values than P_eff_ values estimated based on the reported and calculated P_eff_ values, indicating that permeation through the unstirred water layer was not the rate limiting step of whole membrane permeation. In addition, both drug substances are reportedly classified in the Biopharmaceutical Classification System (BCS) as class II [23, 24], indicating high permeability and low solubility. In particular, the BCS considers drug substances with a P_eff_ greater than 1.5 × 10^–4^ cm/sec as having “high permeability” [44]. Because the estimated P_eff_ of ezetimibe based on the reported P_app_ was lower than the criteria, possibly resulting in deviation from the definition “high permeability”, the PK simulations to analyze the EHC processes of the two drugs were carried out with P_eff_ values estimated using the calculated P_app_.

**Table II:** Summary of estimated permeability rate constants for meloxicam and ezetimibe

	Meloxicam	Ezetimibe
P_eff_ estimated based on reported P_app_ (cm/sec)	3.90 × 10^–4^	1.21 × 10^–4^
P_eff_ estimated based on calculated P_app_ (cm/sec)	3.79 × 10^–4^	3.06 × 10^–4^
P_UWL_ (cm/sec)	8.00 × 10^–4^	7.60 × 10^–4^

The model used in this study assumed that the drugs were distributed to the central compartment, the peripheral compartment, and the gallbladder compartment after intestinal absorption. The post-absorption PK parameters (distribution volume, elimination rate constant, distribution rate constant between the peripheral and central compartments, and distribution rate constant to the gallbladder compartment) of the two drugs were obtained from data estimated in past reports [6, 18]. Of note, the bioavailability of ezetimibe has not been determined to date because it is virtually insoluble in the aqueous phase and cannot be administered via iv [42]. Nevertheless, as ezetimibe has been reportedly classified as BCS class II (high permeable) [44] and the extent of the absorption is not affected by concomitant food [42], we hypothesized that it could be completely absorbed in humans and that the distribution volume divided by oral bioavailability could be considered as the volume divided by the fractions surviving metabolism in the gastrointestinal tract and liver. The distribution volume of meloxicam was estimated using iv data in a previous report [18]. The transit of both drugs from the gallbladder to the small intestine was assumed to follow the following equation:

10 $$\frac{d{GBR}_{t}}{dt}={A}_{GB}\cdot {K}_{GBR}\cdot GBE$$

where *GBR*_*t*_ is the amount of drug already excreted into the intestine at time *t*; *A*_*GB*_ is the amount of drug in the gallbladder compartment; *K*_*GBR*_ is the excretion rate constant from the gallbladder, which was set to a high value (21 h^−1^) in this study to describe rapid excretion (like a bolus excretion) [6]; *GBE* is the coefficient change to 0 (in the overall term when excretion does not happen) or 1 (passage of 0.75 h since meal ingestion) to achieve intermittent excretion within the limited duration of meal timing.

To investigate the effect of the model structure of EHC on the predicted PK profile, we performed PK predictions using the four models below:

- Model 1: EHC was not assumed to happen; the gallbladder was assumed to be an organ for elimination, and the distribution rate constant to the gallbladder was therefore added to the elimination rate constant.
- Model 2: Drugs in the gallbladder were assumed to be directly emptied to the central compartment instead of to the intestinal compartment.
- Model 3: EHC with drug emptying to the intestine was considered, in which the permeation rate constant used in the reabsorption process was the same as that used in the fasted state (namely, *f* = 1 was assumed in Eq. (7)).
- Model 4: This model used the same compartments as Model 3, but the reabsorption of EHC was done with consideration to the free fraction calculated based on the solubility ratio of FeSSIF-V2 to FaSSIF-V2.

The prediction of PK profiles was performed using Stella Professional (isee systems, Lebanon, NH, USA) with a delta time of 0.01 h and the 4th order Runge–Kutta integration method. The predicted PK profiles were compared to the observed PK profiles in humans [32, 45].

Mean Absolute Percentage Error (MAPE) was calculated to consider the difference between each predicted PK and the observed one following the following equation:

11 $$MAPE=\frac{100}{n}\sum_{i=1}^{n}\left|\frac{{P}_{i}-{O}_{i}}{{O}_{i}}\right|$$

where *n* is the number of the sampling points; and *P*_*i*_ and *O*_*i*_ are the predicted and observed plasma drug concentrations, respectively, at each sampling point. MAPE calculation was performed using the plasma concentration data first sampled after each meal ingestion, namely at 4.5, 12. 24, 48 and 72 h for Mobic® tablet and 4.5, 11, 24, 36, 48 and 72 h for Zetia® tablet. In the case that the timing of sampling was the same as that of meal ingestion, the predicted concentration at the timing with added duration (0.1 h) was adopted for calculation, given that EHC was described as occurring at 0.1 h after each meal ingestion.

## Results and Discussion

### Solubility Determination in the Biorelevant Media and Calculation of Fraction of Free Drug

The solubility of meloxicam and ezetimibe was measured in the biorelevant media (FaSSIF-V2 and FeSSIF-V2). The results are summarized in Table III. The solubility of meloxicam in FaSSIF-V2 and FeSSIF-V2 was 0.172 mg/mL and 0.0427 mg/mL, respectively. Meloxicam is a weak acid compound with a pKa of 1.1 and 4.2 [25]. Since the pH of FeSSIF-V2 is 5.8, at which the increase in non-ionic forms of meloxicam is greater than that at the pH 6.5 of FaSSIF-V2, the solubility in FeSSIF-V2 was likely decreased compared with that in FaSSIF-V2. On the other hand, the solubility of this drug in blank FaSSIF-V2 and blank FeSSIF-V2 tested separately was 0.164 mg/mL and 0.0325 mg/mL respectively, which did not differ from the corresponding solubility in media in which the surfactant was included. We therefore considered that the solubilization of meloxicam by the bile micelles was insignificant.

**Table III:** Solubility of meloxicam and ezetimibe in biorelevant media and free fraction of drugs estimated in FeSSIF-V2

	Meloxicam	Ezetimibe
FaSSIF-V2 (mg/mL)	0.172 ± 0.0123	0.00379 ± 0.000133
FeSSIF-V2 (mg/mL)	0.0427 ± 0.00153	0.0223 ± 0.000203
Free fraction	Regard as 1	0.169

The solubility of ezetimibe in FaSSIF-V2 and FeSSIF-V2 was 0.00379 mg/mL and 0.0223 mg/mL, respectively, showing that solubility in FeSSIF-V2 was much higher than that in FaSSIF-V2, although the absolute values were lower than those of meloxicam. This is because the pKa of ezetimibe is 9.75 [42] and the drug occurs mostly in the non-ionic state in the intestine, unlike meloxicam, which can exist in the ionic state in the intestine.

The fraction of free drugs in FeSSIF-V2 was calculated based on the solubility data using Eq. (8). The results are also shown in Table III. Since the solubility of meloxicam in the biorelevant media scarcely increased compared to those in the corresponding blank media, the PK simulation for meloxicam was conducted in Section "Oral PK Prediction for Mobic® Tablet and Zetia® Tablet using M&S with Consideration to EHC" without consideration of the solubilization in micelles – in other words, the free fraction of this drug was assumed to be 1 even in the fed small intestine.

### *In Vitro* Dissolution Profiles of Mobic® Tablet and Zetia® Tablet in the Biorelevant Media

**Fig. 3:**
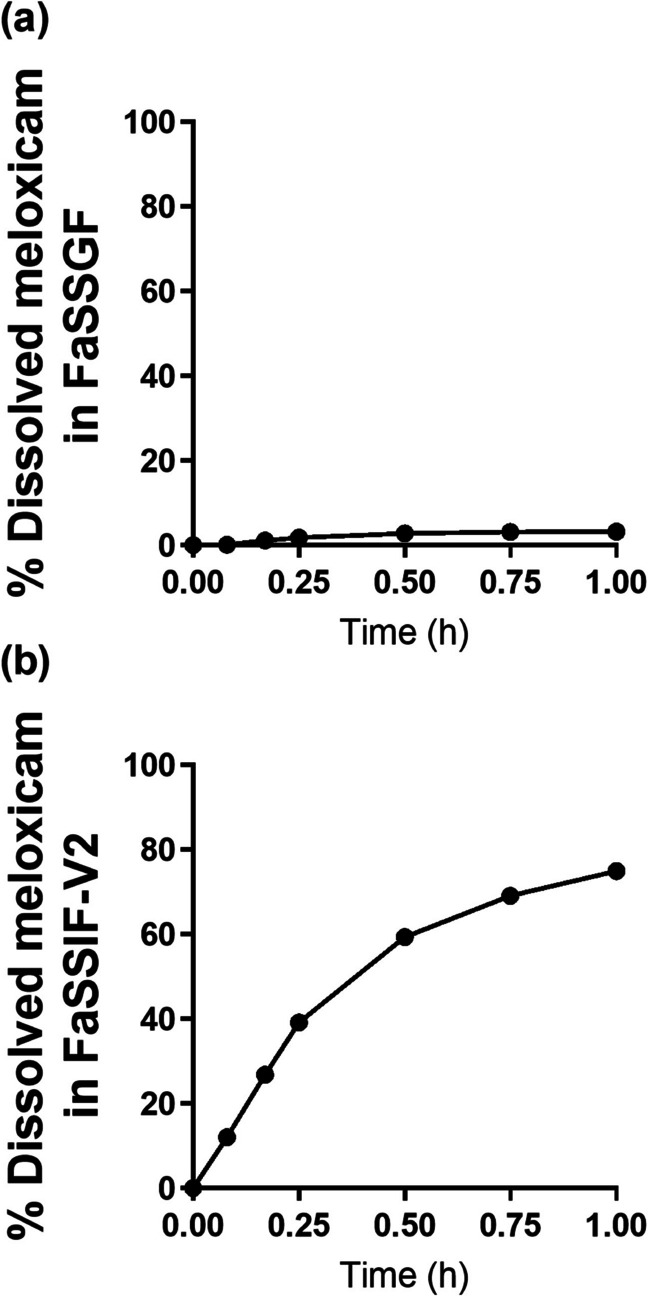
Dissolution profiles of meloxicam from Mobic.® tablet in (**a**) FaSSGF and (**b**) FaSSIF-V2.

**Fig. 4:**
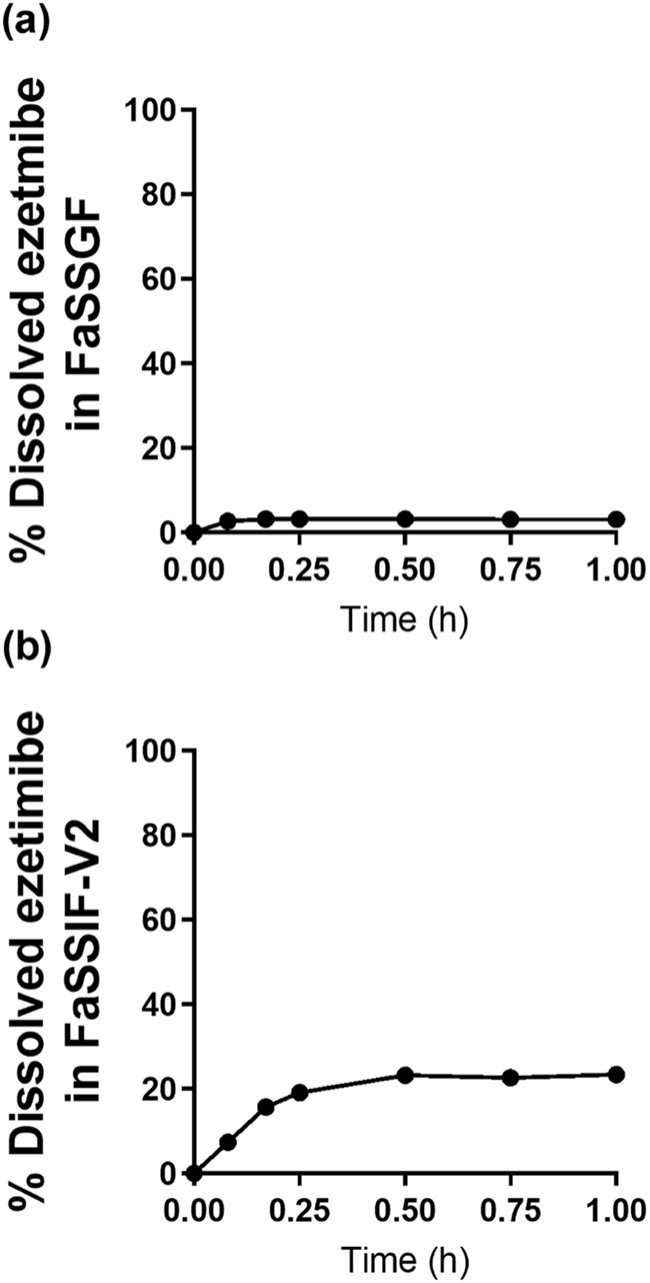
Dissolution profiles of ezetimibe from Zetia.® tablet in (**a**) FaSSGF and (**b**) FaSSIF-V2.

To estimate the dissolution profiles of Mobic® tablet and Zetia® tablet in the gastrointestinal tract (GIT), *in vitro* dissolution testing in FaSSGF and FaSSIF-V2 was conducted. The *in vitro* dissolution profiles of Mobic® tablet and Zetia® tablet are shown in Figs. 3 and 4, respectively. Both tablets showed almost no dissolution in FaSSGF since both compounds existed as the non-ionic forms in the gastric pH, based on pKa values. Although dissolution of meloxicam up to about 80% was seen in FaSSIF-V2, the dissolution of ezetimibe in this medium was only around 20%, with the difference likely attributable to the respective amount occurring in the ionic state at a neutral pH. To solve this low solubility of ezetimibe in the media, a variety of formulation technologies have been applied, although these have not been applied for the Zetia® tablet [23, 46, 47].

The solubility and dissolution rate constants of both tablets for the PK simulation are summarized in Table IV. Their solubility in FaSSGF was adopted from the plateau of the dissolution profile. The solubility of ezetimibe in FaSSIF-V2 slightly differed depending on the measurement method, namely 0.00493 mg/mL using from the infinity point of the dissolution test of the tablet and 0.00379 mg/mL using the drug substance in the solubility test (shown in Table II). Zetia® tablet contains sodium dodecyl sulfate, and the apparent solubility of ezetimibe likely increased temporarily. The solubility of ezetimibe in Zetia® tablet measured in the dissolution test of FaSSIF-V2 was finally adopted for the M&S. By contrast, for Mobic® tablet in FaSSIF-V2, the solubility data in Table II was adopted as the solubility of meloxicam in the drug product because complete dissolution was observed on further dissolution testing with a paddle revolution speed of 250 rpm following testing for 60 min at 50 rpm. The dissolution rate constants of Mobic® tablet were compensated using Eq. (2) since the dose for the *in vitro* dissolution test differed from that used in the PK study in humans.

**Table IV:** Solubility and dissolution rate constant of Mobic® tablet and Zetia® tablet for the modified Noyes-Whitney equation

		Meloxicam (Mobic® tablet)	Ezetimibe (Zetia® tablet)
Solubility (mg/mL)	FaSSGF	0.00121	0.00106
	FaSSIF-V2	0.172	0.00493
Dissolution rate constant (mL mg ^−2/3^/h)	FaSSGF	0.739	4.75
	FaSSIF-V2	0.0350	1.24

### Oral PK Prediction for Mobic® Tablet and Zetia® Tablet using M&S with Consideration to EHC

The oral PK profiles of Mobic® tablet and Zetia® tablet were first predicted using M&S with consideration to EHC, then compared to each of the observed PK profiles. Simulation conditions are detailed in Section "*In silico* Modeling and Simulation".

**Fig. 5:**
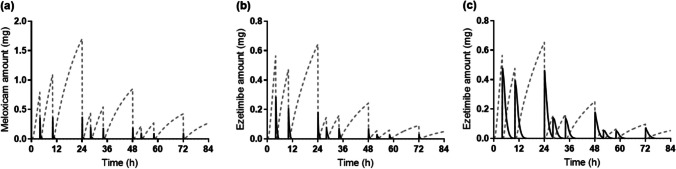
Simulated drug amount in the gallbladder compartment (*gray dash line*) and fed intestinal compartment (*black solid line*) for meloxicam in Model 3 (**a**), and for ezetimibe in Model 3 (**b**) and Model 4 (**c**).

Understanding the kinetics of the drug amount in the intestinal and gallbladder compartments are especially important in this study. Figure 5 shows the profile of the simulated drug amount in the gallbladder compartment and the intestinal compartment of the fed state for each drug. The accumulation pattern of meloxicam (Fig. 5a) and ezetimibe (Fig. 5b and c) in the gallbladder compartment was similar due to the same setting for the gallbladder emptying rate constant and the duration for both drugs. However, the profile of the drug amount in the intestinal compartment differed in the models. Meloxicam (Fig. 5a) and ezetimibe (Fig. 5b) simulated with Model 3, which did not consider the fraction of micelles, showed rapid reabsorption and almost no accumulation in the fed intestinal compartment even after each gallbladder emptying. In contrast, ezetimibe simulated with Model 4 in consideration of micellization (Fig. 5c) showed greater accumulation in the fed intestine after gallbladder emptying compared to that simulated with Model 3. This is likely because the permeability rate was decreased by consideration of micellization in the reabsorption process in the fed intestine in Model 4.

**Fig. 6:**
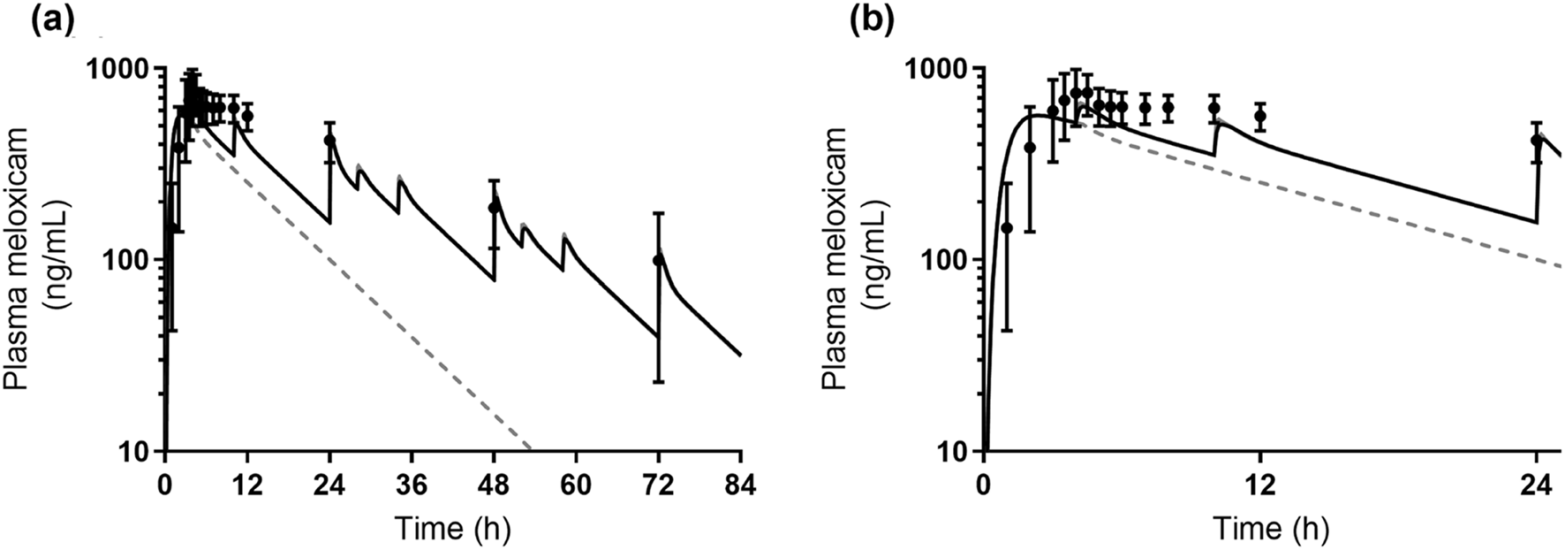
Observed (*black closed circles*) and predicted PK profiles considering EHC (*black solid line*, Model 3), without considering EHC (*gray dash line,* Model 1), and considering EHC while the drugs were assumed to be directly emptied into the central compartment (*gray solid line*, Model 2) for Mobic® tablet. PK profiles were simulated assuming that EHC happened for 72 h (and did not happen after 72 h). Observed PK profile is shown as mean ± SD. (**a**) shows the whole PK profile and (**b**) is the enlarged profile over 24 h.

**Fig. 7:**
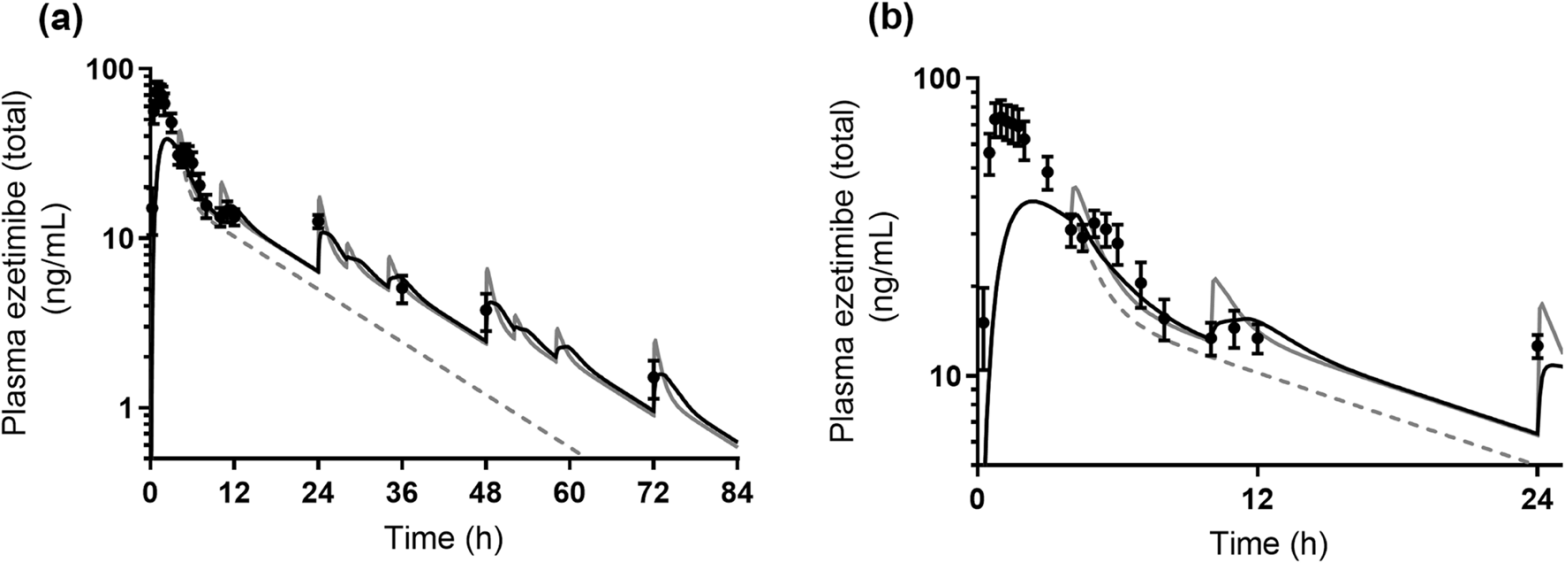
Observed (*black closed circles*) and predicted PK profiles considering EHC (*black solid line*, Model 4), without considering EHC (*gray dash line,* Model 1), and considering EHC while the drugs were assumed to be directly emptied into the central compartment (*gray solid line*, Model 2) for Zetia® tablet. PK profiles were simulated assuming that EHC happened for 72 h (and did not happen after 72 h). Observed PK profile is shown as mean ± SD. (**a**) shows the whole PK profile and (**b**) is the enlarged profile over 24 h.

**Fig. 8:**
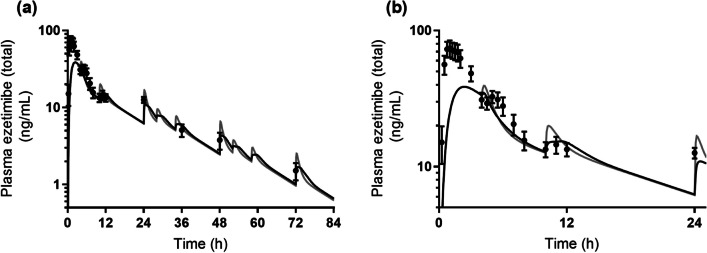
Observed (*black closed circles*) and predicted PK profiles using the same P_eff_ as in the fasted state (*gray solid line*, Model 3) or the P_eff_ compensated by the free fraction estimated based on the solubility ratio of FeSSIF-V2 to FaSSIF-V2 (*black solid line,* Model 4) for Zetia® tablet. PK profiles were simulated assuming that EHC happened for 72 h (and did not happen after 72 h). Observed PK profile is shown as mean ± SD. (**a**) shows the whole PK profile and (**b**) is the enlarged profile for 24 h.

The predicted PK profiles with consideration to EHC (*black solid line*, Model 3 or Model 4), without consideration to EHC (*gray dash line*, Model 1), and with consideration to EHC while the drugs were assumed to be directly emptied into the central compartment (*gray solid line*, Model 2) are shown in Figs. 6 and 7 for Mobic® tablet and Zetia® tablet, respectively. In addition, the simulations for ezetimibe are also shown in Fig. 8 to consider the impact of micellization on the reabsorption process in the prediction. MAPE values were also calculated to consider the difference between the respective predicted and observed PKs; results are summarized in Table V for both tablets.

**Table V:** MAPE values of Mobic® tablet and Zetia® tablet

	MAPE values			
	Model 1	Model 2	Model 3	Model 4
Mobic® tablet	70.9%	14.5%	13.5%	13.5%
Zetia® tablet	47.9%	36.0%	21.6%	15.9%

When EHC was not considered (Model 1), the MAPE values of Mobic® tablet and Zetia® tablet were 70.9% and 47.9%, respectively, due to significant overestimation of the elimination rate of the drugs compared to the actual PK profiles. Predictions with direct EHC emptying to the central compartment (Model 2) showed MAPE values of 14.5% and 36.0% for Mobic® tablet and Zetia® tablet, respectively. Some reports have adopted direct emptying from the gallbladder to the blood circulation to describe the EHC [4, 8, 20, 21]. Since the drugs used in the present study showed relatively high MAPE values when described by Model 2, it is possible that the feasibility of this simple description depended on the properties of compounds. The description of Model 2 could be assumed to imitate the situation in which the compounds were ultimately rapidly absorbed. Therefore, the higher the permeation rate constant the compound has in the small intestine, the smaller the impact this simple description is likely have on prediction accuracy.

The description to empty the drug into the GIT without compensating for the permeation rate constant (Model 3) showed MAPE values of 13.5% and 21.6% for Mobic® tablet and Zetia® tablet, respectively. In addition, the description of Model 4 with compensation showed a MAPE of 15.9% for Zetia® tablet, which was lower than the value with Model 3. Ezetimibe was clearly shown to be solubilized by the mixed micelles in the *in vitro* study (Table III). Further, it was revealed that PK prediction of Zetia® tablet without consideration of micelle solubilization in the reabsorption process showed a higher MAPE value. By contrast, since meloxicam was not entrapped in the micelles and solubilized in the *in vitro* study (Table III), MAPE values for this drug will be the same between Models 3 and 4. Therefore, for compounds which are solubilized by micelles in the fed state, more accurate estimation of the plasma concentration after EHC may require compensation for the permeation rate constant based on the amount of free fraction. The higher the plasma concentration peaks of a compound after EHC, the absolutely bigger the impact of compensation of the permeation rate constant on prediction accuracy.

The duration and the rate (constant) should be particularly considered in describing the gallbladder emptying process. As a premise, the duration and rate which determine how gallbladder emptying happens are considered physiological parameters that are independent of the kind of drug. In accordance with a past report relating M&S for ezetimibe [6], we set the value to 0.75 h as duration to conduct the analysis. This appears a reasonable value in terms of physiological conditions. In the gallbladder emptying process, drugs accumulated in the gallbladder are assumed to be emptied into the duodenum in a bolus manner with bile acids at the time of meal ingestion. To describe this bolus excretion, a high value for the gallbladder emptying rate constant was combined with a switch function which make the emptying process work in a fixed duration. This type of description has been used in past reports for ezetimibe M&S [6], in which 21 h^−1^ was used as the rate constant. We used this value as the basic gallbladder emptying rate constant for simulations (Figs. 6, 7 and 8). In this regard, as described in a past study, emptying of drugs from the gallbladder in a bolus manner can be done by arbitrarily setting a high positive value. Another high positive value—67.5 h^−1^—has been also reported [48]. On use of this value in the present study for ezetimibe, Model 4 with compensation showed a MAPE value of 11.9%, which was much lower than the 32.3% predicted by Model 3 without compensation.

Further improvement in the description of the first absorption phase of drugs in this study may be possible (Figs. 6b and 7b). An alternative approach to describing the absorption phase has recently been reported. Called the Finite Absorption Time (FAT) concept [49, 50], the kinetics of membrane permeation in this concept are described using a zero-order equation calculated with the absorbable fraction (like dissolved fraction) in the lumen divided by a finite time for absorption. Various combinations of input rate and duration finally lead to the most appropriate description for the absorption process. Nevertheless, we decided to use first order kinetics, depending on drug concentration in the lumen for absorption. This is because our investigation particularly focused on the necessity of compensation for the permeability rate constant in the reabsorption process of EHC with consideration to drug entrapment in bile micelles, which cannot be described using a zero-order equation.

In this M&S for EHC, we hypothesized that a bolus of highly concentrated bile acid and drug would be emptied together into the duodenum upon the stimulus of the meal, where the drug could then be entrapped in bile micelles. This explains why the extent of solubilization (entrapment) by the micelles was estimated based on the *in vitro* solubility tests. By contrast, in an actual GIT after meal ingestion, not only bile micelles but also emulsions containing dietary lipids could also form. Given that bile acid concentration in the GIT in the fed state is much higher than the CMC [13], the mixed micelles would greatly contribute to the reabsorption process of drugs and fat components through the small intestinal epithelium cells. Accordingly, we did not consider the influence of emulsions on the reabsorption process in this study.

Ezetimibe is reported to be extensively metabolized in the intestinal mucosa and the liver through the absorption process. The main metabolite is a glucuronic acid conjugate. Since not only the parent drug but also the metabolite has a pharmacological function (inhibitory effect on cholesterol absorption) in the intestine [51], consideration of the metabolite PK for this drug is also markedly important. In this regard, bioequivalence studies of Zetia® tablet in humans considered not only individual concentrations of the parent drug and the metabolite but also their total concentration [32, 52]. Some reports have constructed an M&S describing compartments for both parent drugs and metabolites [20], while others have constructed an M&S with their total amount [6]. In this regard, the plasma concentration of glucuronide is about seven-fold higher than that of the parent drug in terms of AUC [32]. Since the T_max_ of glucuronide is also more rapid than that of the parent drug, metabolism rate in enterocytes and hepatocytes appears to be very rapid. Accordingly, the metabolic processes might not be rate limiting steps in determining the distribution and PK in the body. Therefore, in our present study, to reduce the complexity of parameters in the simulation while considering the reabsorption process, we described the PK for the total amount of both parent drug and metabolite.

The glucuronide of ezetimibe is considered to undergo deconjugation by β-glucuronidase from the intestinal bacteria after excretion into the duodenum [53]. Facultative anaerobic bacteria which produce this enzyme are widely present in the upper intestine, including the duodenum and jejunum. It has been reported that *Bifidobacterium adolescentis* and *Lactobacillus acidophilus*, which are classified as facultative anaerobic bacteria, almost completely hydrolyze genistein and daidzein, types of soy isoflavone glycoside [54]. In fact, on oral administration in humans, genistein and daidzein were completely detected in plasma as aglycones, the hydrolysate form of the glucuronide [55]. This is why we considered that the glucuronide of ezetimibe would be very rapidly deconjugated by the bacteria in the upper intestine, where permeation through the intestinal membrane is highly likely to be the rate limiting step over the reabsorption process and may be described based on the characteristics of the parent drug. Although meloxicam is reportedly metabolized by CYP2C9 and also partly by CYP3A4 in the liver [56], this drug exists mainly as the parent drug in plasma [57]. Since the past PK studies of meloxicam measured only the parent drug [18], we constructed the M&S for Mobic® tablet by describing the plasma concentration of the parent drug in the present study.

Some methods have been reported to estimate the extent of entrapment of drugs inside micelles [58, 59]. The methods using solubility data in this study can be relatively easily applied to estimating micellization without any special simulations. It was predicted in advance that ezetimibe was likely entrapped in micelles since its pKa information indicated that it exists completely as non-ionic forms at the neutral pH of the intestine in the fed state. In contrast, meloxicam—an acidic compound with pKa 4.5—was predicted to be less solubilized by micelles since it exists as ionic forms at neutral pH. The actual solubility data supported this expectation. Therefore, the necessity for compensation of the permeability rate constant in the M&S of drugs undergoing EHC can be roughly estimated based on the characteristics of the drug, and implementation of the experiment could also be considered accordingly.

A single point comparison alone between the observed and predicted plasma concentration is unlikely to be adequate in considering the prediction accuracy of these models. The MAPE calculation enables us to consider the prediction results with the data for multiple sampling points. Since the purpose of this study was to investigate the impact of each model on the PK prediction of drugs undergoing EHC, MAPE calculation was done using plasma concentrations at the timing of EHC, rather than earlier sampling points (such as C_max_). If the data at earlier sampling points had been included in the MAPE calculation, the MAPE value—especially in the case of Zetia® tablet—would have been increased in view of the observed and predicted PK profiles (Figs. 7 and 8). Even in that case, however, the rank order of MAPE values among the models would not have changed, because the initial absorption process before EHC was described using the same compartments and variables for each model. The sampling points chosen for MAPE calculation in this study therefore appear suitable for the investigation of differences among the models.

## Conclusion

In the present study, we constructed for the first time a model to predict the PK profile of compounds undergoing EHC in humans, which took account not only of the transit and dissolution of the formulation but also the reabsorption process in consideration of the fed state of the GIT. Meloxicam and ezetimibe were selected as model drugs. *In vitro* solubility testing soon clarified that ezetimibe was solubilized by bile micelles whereas meloxicam was not. When the PK profiles of ezetimibe in Zetia® tablet was predicted with compensation for P_eff_ based on the free fraction of the drug estimated by the *in vitro* solubility test (Model 4), the MAPE value was lower than that in Model 3, where the effect of micellization on P_eff_ was not considered. By contrast, meloxicam in Mobic® tablet, which was not solubilized by bile micelles, showed a low MAPE value even without compensation. We concluded that for drugs which undergo EHC and are solubilized by bile micelles, it appears that the accuracy of PK prediction is improved by describing the reabsorption process using the permeability rate constant with compensation based on the free fraction of drugs. Given the limited number of drugs sampled in this study, further investigation with a wider range of compounds will provide more conclusive results.
